# Microwave-Assisted Synthesis of 5′-*O*-methacryloylcytidine Using the Immobilized Lipase Novozym 435

**DOI:** 10.3390/molecules27134112

**Published:** 2022-06-26

**Authors:** Sany Chea, Khac Toan Nguyen, Ruben R. Rosencrantz

**Affiliations:** 1 Fraunhofer Institute of Applied Polymer Research, Biofunctionalized Materials and (Glyco) Biotechnology, Geiselbergstr. 69, 14476 Potsdam, Germany; sany.chea@iap-extern.fraunhofer.de (S.C.); toan.nguyen84@gmx.de (K.T.N.); 2 Chair of Polymer Materials and Polymer Technologies, Institute of Chemistry, University of Potsdam, Karl-Liebknecht-Str. 24–25, 14476 Potsdam, Germany; 3 Institute of Chemistry, Technical University of Berlin, Str. des 17. Juni 115, 10623 Berlin, Germany

**Keywords:** microwave irradiation, Novozym 435, cytidine, monomer, smart materials

## Abstract

Nucleobase building blocks have been demonstrated to be strong candidates when it comes to DNA/RNA-like materials by benefiting from hydrogen bond interactions as physical properties. Modifying at the 5′ position is the simplest way to develop nucleobase-based structures by transesterification using the lipase Novozym 435. Herein, we describe the optimization of the lipase-catalyzed synthesis of the monomer 5′-*O*-methacryloylcytidine with the assistance of microwave irradiation. Variable reaction parameters, such as enzyme concentration, molar ratio of the substrate, reaction temperature and reaction time, were investigated to find the optimum reaction condition in terms of obtaining the highest yield.

## 1. Introduction

Nucleobase-bearing compounds are gaining attention in Materials Science by mimicking DNA/RNA structures. Strong base-pairing properties of complementary nucleobases adenine with thymine and cytosine with guanine are the fundamental building block for the development of smart programmable materials [1,2,3,4,5]. In particular, thermoresponsitivity can be targeted since hydrogen bond interactions are labile when exposed to heat [6]. The synthesis of nucleobase-based polymers requires challenging isolation of their monomers. Low solubility in nucleobases or reactive functional groups of nucleosides and especially, of integrated ribose require the development of a multi-step synthesis, including protection and deprotection steps [7,8,9]. Applying enzymes as biocatalysts greatly facilitates these syntheses due to their regioselectivity and specificity [10,11]. The tedious protection–deprotection methods for hydroxyl groups can be avoided to reduce the synthesis steps. The opportunity for a one-step functionalization is not the only advantage; it is also beneficial in terms of “green chemistry”. Enzymes are highly efficient, even under mild conditions, such as low temperature and low pressure. After proper work-up, they can even be reused in several synthesis cycles [12].

The industrial application of the enzyme class lipases (EC 3) ranges from the detergent to the pharmaceutical industry [13,14]. Their biological function is focused on the hydrolysis of lipids. This hydrolysis is an equilibrium reaction, and depending on the reaction condition, the reverse reaction, (trans)esterification, may be favored [15]. Due to their high selectivity and specificity towards functional groups, lipases are a good choice for introducing polymerizable groups into nucleosides for further thermoresponsive material designs. Uridine was esterified using the non-commercially available methacrylocylacetone oxime and the lipase Novozym 435, a commercially available isoform B of Candida antarctica (Cal B) lipase with a microporous acrylic polymer resin, which comes with a spherical pearl morphology. The yield was 83% after a reaction time of 52.5 h. Complementary adenine yielded in 44% [16]. Based on these results, cytidine was functionalized with a polymerizable group. The isolation required a reaction time of 22 h and resulted in a yield of 47%. Synthesis of the complementary guanosine-based monomer using the lipase Novozym 435 was only feasible with time-consuming protection–deprotection steps [11]. 

Over the last 30 years, the application of microwave irradiation to chemical reactions has increased significantly. It is used in organic [17,18,19] and organometallic synthesis [20,21], inorganic solid–state reactions [22], catalysis and even peptide synthesis [23,24], polymer chemistry or nanotechnology [25]. Due to the steady heat distribution, the yield in organic syntheses can be increased in a reduced reaction time compared to conventional heating with an oil bath. Combining microwave irradiation with enzyme catalysis is a common method for the synthesis of compounds [26,27,28]. The application of a biocatalyst enables derivatization of carbohydrate moieties without the use of protecting groups, while microwave irradiation significantly increases reaction kinetics, as reflected in reaction time and/or reaction yield. This synergy of microwave irradiation and enzymatic catalysis for derivatization of carbohydrate structures was revealed. In the past, the enzymatic conversion of methyl α-ᴅ-glucosides with dodecanoic acid was studied. Compared to conventional heating, the assistance of microwave irradiation increased the yield from 55% to 95%, at a reaction temperature of 95 °C [29]. The synthesis of sorbitan methacrylate was also optimized using a combination of the biocatalyst and microwave radiation [30].

**Figure 1:**
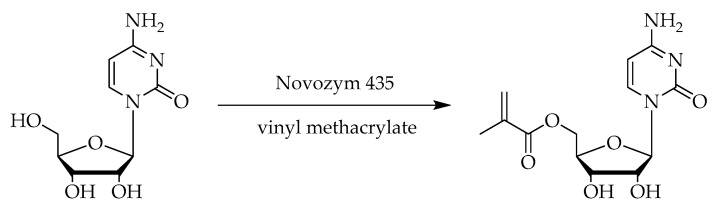
Enzyme-catalyzed synthesis of 5′-*O*-methacryloylcytidine.

This work focused on the optimization of a lipase-catalyzed synthesis of cytidine-based monomer for further programmable material designs under microwave irradiation (Figure 1). The use of lipase makes it possible to minimize the synthesis of such elaborately synthesized cytidine monomers to a single synthesis step. The combination with microwave irradiation accelerates the reaction rate and is, therefore, more efficient due to a reduced reaction time. The lipase Novozym 435 enables functionalization of the 5′-hydroxyl group without protection of the remaining hydroxyl groups. The influence of synthesis parameters was analyzed to determine the optimal reaction conditions. Varying reaction parameters, such as enzyme concentration, substrate, molar ratio, reaction temperature and reaction time, were analyzed to determine optimal reaction conditions. 

## 2. Materials and Methods

### 2.1. Materials and Instrumentation

#### 2.1.1. Chemicals

All reagents and solvents were used without further purification. Cytidine (99%) was purchased from Carbosynth (Berkshire, UK), 1,4-dioxane (>99.5%) from Carl Roth (Karlsruhe, Germany). Novozym 435 (Lipase B from Candida antarctica, immobilized on acrylic resin, >5000 U/g), vinyl methacrylate (98%), methyl methacrylate (99%) and 2,6-di-tert-butyl-4-methylphenol (BTH, >99%) were received from Sigma Aldrich (St. Louis, MO, USA). The solvents for chromatographic purification acetonitrile (>99.95%), ethyl acetate (>99.8%) and methanol (>98.5%) were from VWR (Radnor, PA, USA). Deuterated solvents D_2_O (99.9%) and DMSO-d_6_ (99.8%) were obtained from Deutero (Kastellaun, Germany).

#### 2.1.2. Instrumentation

ESI MS spectra were recorded with a Perkin Elmer Flexar SQ 300 MS Detector. Samples were prepared by solving in an acetonitrile/water (1:1) mixture with additional formic acid (0.1%) and measured at a temperature of 300 °C with a flow rate of 15 µL min^−1^. Nuclear magnetic resonance (NMR) spectroscopy was performed with a Bruker AVANCE NEO (400 MHz) spectrometer. Deuterated solvents were used as standards. Chemical shifts are given in the δ-scale in ppm relative to solvent peaks. Multiplicities are displayed with the coupling constants in Hertz (Hz).

### 2.2. Enzymatic Transesterification

#### 2.2.1. Conventional Heating

5′-*O*-methacryloylcytidine was prepared conventionally using a modified synthesis [31]. Briefly, cytidine (50.3 mg, 0.207 mmol, 1 eq) was suspended in 1,4-dioxane (2 mL), following the addition of a catalytic amount of BTH, vinyl methacrylate (0.740 mL, 0.616 mmol, 3 eq) and Novozym 435 (0.277 mg, 5.5 wt%). The reaction mixture was stirred overnight under N_2_ atmosphere at 60 °C. After reacting over 22 h, the reaction mixture was concentrated and purified using column chromatography (SiO_2_, 7:3 EtOAc/MeOH). The desired compound was isolated as a white solid (11.0 mg, 35.2 µmol) with a yield of 16.9%. 

^1^H NMR (D_2_O, 400 MHz, rt): δ [ppm] = 7.67 (d, ^3^*J* = 7.6 Hz, 1H, H-17); 6.14 (s, 1H, CH_2_(21)); 5.99 (d, ^3^*J* = 7.5 Hz, 1H, H-16); 5.87 (d, ^3^*J* = 2.6 Hz, 1H, H-8); 5.77 (s, 1H, CH_2_(21)); 4.59 (dd, *J* = 12.6 Hz, ^3^*J* = 2.2 Hz, 1H, CH_2_(2)); 4.45 (dd, *J* = 12.6 Hz, ^3^*J* = 4.1 Hz, 1H, CH_2_(2)); 4.28–4.38 (m, 3H, H-3, H-6, H-4); 1.94 (s, 3H, CH_3_(22)).

^13^C NMR (D_2_O, 400 MHz, rt): δ (ppm) = 168.98 (C=O(4)); 166.06 (C-NH_2_(12)); 157.29 (C=O(13)); 141.02 (C-H(10)); 135.44 (C-(3)); 127.29 (CH_2_(2)); 96.04 (C-H(11)); 90.77 (C-H(9)); 80.81 (C-H(6)); 74.05 (C-H(8)); 69.11 (C-H(7)); 63.34 (CH_2_(5)); 17.45 (CH_3_(1)).

#### 2.2.2. Microwave Irradiation

The used multimode microwave reactor was a START microchemist 1500 from MLS GmbH. The warmup time was set to 5 min to reach the desired temperature and, the maximum power to 1000 W for the whole synthesis procedure. Actual reaction temperature and reaction time were varied as followed as specified in experiments below.

#### 2.2.3. Choice of Substrate

Cytidine (100 mg, 0.414 mmol) was impregnated with Novozym 435 (12.7 wt%) by stirring the lipase with an aqueous solution of cytidine. After freeze drying, vinyl methacrylate (1.50 mL, 14.5 mmol, 1:35) or methyl methacrylate (1.37 mL, 14.5 mmol, 1:35) was added to the reaction mixture with BHT (150 µg, 0.681 µmol). The reaction mixture was then stirred at 95 °C for 30, 95 and 120 min under microwave irradiation and purified using a preparative HPLC device (reverse phase C_18_ silica, 15% acetonitrile in H_2_O).

#### 2.2.4. Effect of Enzyme Concentration

An aqueous solution of cytidine (100 mg, 0.414 mmol) was stirred with Novozym 435 with either 5.5, 12.7 or 22.5 wt%. To impregnate, this cytidine Novozym 435 mixture was stirred at room temperature for 10 min, followed by drying. Afterwards, vinyl methacrylate (1.50 mL, 14.5 mmol, 1:35) and BHT (150 µg, 0.681 µmol) were added to the reaction mixture in a microwave vessel at 95 °C for 30, 60 and 120 min. The desired compound was isolated after purifying using a preparative HPLC device (reverse phase C_18_ silica, 15% acetonitrile in H_2_O).

#### 2.2.5. Effect of Reaction Temperature and Reaction Time

To Novozym 435 (12.7 wt%) -impregnated cytidine (100 mg, 0.414 mmol), BHT (150 µg, 0.681 µmol) and vinyl methacrylate (1.50 mL, 14.5 mmol, 1:35) were added. The reaction mixture was radiated with microwaves for 30 min, 60 min and 120 min at reaction temperatures of 45, 60, 95 and 120 °C, respectively. The product was yielded by a preparative HPLC device (reverse phase C_18_ silica, 15% acetonitrile in H_2_O).

#### 2.2.6. Effect of Molar Ratio

To investigate the excess of the substrate, two different molar ratios to the starting material were used. Therefore, cytidine (100 mg, 0.414 mmol) was impregnated with Novozym (12.7 wt%) before reacting with BHT (150 µg, 0.681 µmol) and vinyl methacrylate with a molar ratio of either 1:35 or 1:76 to cytidine under microwave irradiation at 95 °C for 30, 60 and 120 min. Purification of the reaction mixture occurred with a preparative HPLC device (reverse phase C_18_ silica, 15% acetonitrile in H_2_O).

#### 2.2.7. Recyclability

To examine the recyclability of Novozym 435 after the performed synthesis, the enzyme was filtered and washed with 100 mL of H_2_O. After freeze drying, Novozym 435 was used for recyclability tests. Cytidine (100 mg, 0.414 mmol) was impregnated with recycled Novozym 435 (12.7 wt%). Afterwards, vinyl methacrylate (1.50 mL, 14.5 mmol, 1:35) and BHT (150 µg, 0.681 µmol) were added. The mixture was reacted under microwave irradiation for 30 min at 95 °C, before purifying with a preparative HPLC device (reverse phase C_18_ silica, 15% acetonitrile in H_2_O).

## 3. Results and Discussion

### 3.1. Synthesis Approach and Regioselectivity

Cytidine was functionalized with the polymer-bound lipase Novozym 435 and a suitable transesterification substrate to obtain a polymerizable compound via a one-step synthesis for future development of smart programmable materials. As preliminary trials showed an increase in yield after impregnation of cytidine with Novozym 435, this is necessary to maintain a high yield. To increase the reaction rate, microwave radiation was applied. Due to the consistent heating, in which the molecules are set in motion by vibration and rotation, the addition of microwave radiation increases the energy transfer compared to conventional heating. Since the choice of substrate, enzyme concentration, reaction temperature, reaction time and substrate concentration are decisive factors for the yield, the effects of these parameters are described in more detail in the next chapters. The addition of the radical inhibitor BHT in a catalytic amount is intended to prevent premature polymerization under such harsh conditions of heat and/or microwave irradiation. The conversion rate of an enzymatic (trans)esterification depends on the polarity of the used solvent. The higher the log *p* value, the higher the yield. Hydrophilic solvents, defined by lower log *p* values, showed reduced enzyme activity [32]. Polar solvents, such as DMF, may deactivate enzymes. Nevertheless, apolar solvents are not able to dissolve polar substrates. Previous work showed a peak in conversion rate when using hydrophobic solvents like hexane. First, the yield improved with increased hexane concentration, but depicted a turning point after higher hexane amounts [33]. Evaluation of microwave-assisted lipase-catalyzed reactions in different solvents indicated that the highest conversion yield was achieved when avoiding typical organic solvents. The advantage of using a solvent-free system or using the substrate as the solvent is that the polarity of the substrate is not affected. Therefore, the synthesis with the substrate as solvent was strived for in this work. The yield was determined gravimetrically after chromatographic purification, either in a solvent mixture of ethyl acetate and methanol for column chromatography or a mixture of acetonitrile and water for preparative HPLC. According to the literature described, similar molecular derivatives were isolated with the solvents mentioned and, therefore, after some TLC analyses, they were also used for our purpose. After screening various reaction parameters, a yield of 36.2% was obtained after a reaction time of 30 min at 95 °C with a vinyl methacrylate molar ratio of 1:35 and an enzyme concentration of 12.7 wt%.

To ensure isolation of the correct product, 5′-*O*-methacryloylcytidine was synthesized by conventional heating, according to the literature. Compared to the reaction experiments with microwave irradiation, a molar ratio of the substrate vinyl methacrylate of only 1:3 was used and additionally, 1,4-dioxane as solvent. After a reaction time of 22 h at 60 °C and purification by column chromatography, the desired product was isolated with a yield of 16.9%. 

The advantage of the regioselectivity of enzymes is especially useful in sugar chemistry, which eliminates the need for protection and deprotection steps [10,29,34]. Due to less steric hindrance, primary hydroxyl groups are favored for the interaction compared to the remaining hydroxyl groups. In our case, the C5 position is targeted. As such, 2D NMR spectroscopy analysis in DMSO of the isolated compound revealed the cytidine derivatization of the 5′-OH functional group, indicating an absolute regioselectivity (see Supplementary Materials). 

### 3.2. Choice of Substrate

**Figure 2:**
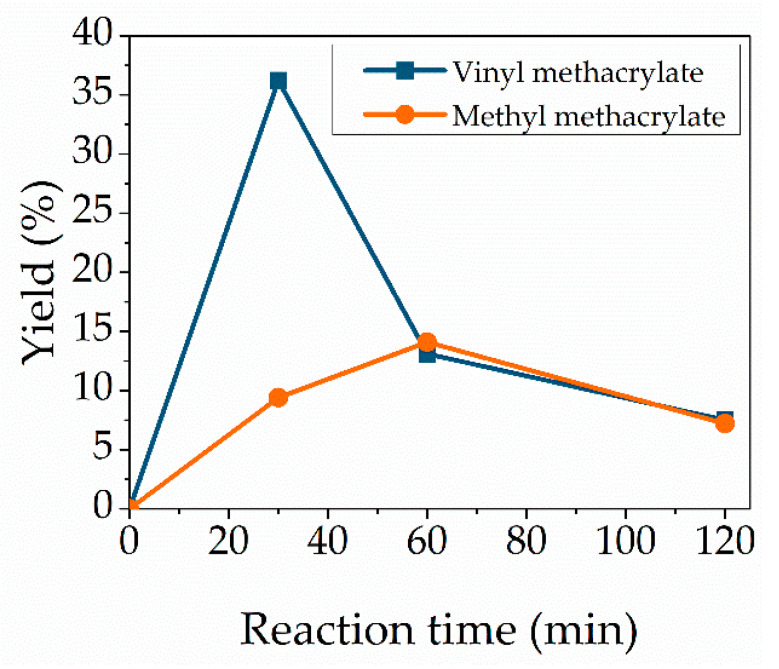
Effect of the choice of the substrate. Reaction conditions: cytidine (100 mg, 0.414 mmol), Novozym 435 (12.7 wt%), 95 °C, 1:35 vinyl methacrylate (blue) or methyl methacrylate (orange). Lines are included as a guide to the eye.

The choice of a suitable substrate must not be neglected, as the type of acyl donors influences the reaction. We selected vinyl methacrylate for the synthesis of the cytidine-based monomer by transesterification based on the following. Since enzymatic transesterification is a reversible reaction, the acyl donor selected should be chosen wisely to shift the equilibrium on the desired reaction side. Compared to alkyl esters, the use of vinyl esters provides better leaving groups, since resulting vinyl alkoxides can stabilize the negative charge more easily than resulting alkyl alkoxide leaving groups [35]. In addition, unlike alkyl esters, transesterification with vinyl esters is irreversible because the resulting leaving group rapidly tautomerizes to a non-nucleophilic carbonyl species. In this case, the leaving group is vinyl alcohol, which is a weak nucleophile and, thus, less involved in reverse reactions. Rapid tautomerization of vinyl alcohol to acetaldehyde leads to irreversibility of transesterification. This leads to increased reaction rates compared to alkyl esters and, thus, also to acid equivalents, such as methacrylic acid, confirmed in previous studies [30,36]. In our case, the first experiments with methyl methacrylate also showed decreased reaction yields compared to vinyl methacrylate. Due to these, vinyl methacrylate was used for further investigations. The highest yield could be observed with a reaction time of 30 min but there were decreases in the case of longer reaction times. This decrease is unusual compared to the reported literature, wherein the yield reaches a plateau. To check whether this occurrence is due to the substrate, microwave-assisted reactions were performed additionally with methyl methacrylate. Here, the same trend can be observed: the yield decreases in terms of long reaction times of 2 h, except that the highest achieved yield is shifted to 1 h compared to the reaction with vinyl methacrylate at 30 min (Figure 2).

### 3.3. Enzyme Concentration

**Figure 3:**
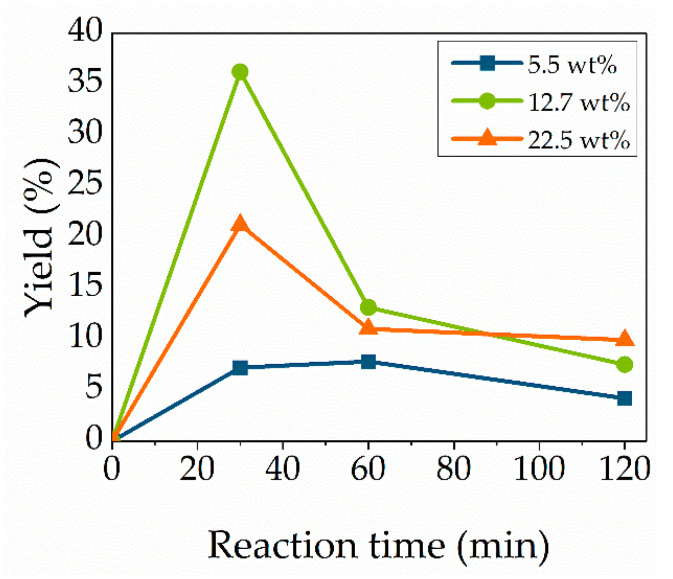
Effect of enzyme concentration. Reaction conditions: cytidine (100 mg, 0.414 mmol), 1:35 molar ratio vinyl methacrylate, 95 °C and Novozym 435 5.5 wt% (blue), 12.7 wt% (green) or 22.5 wt% (orange). Lines are included as a guide to the eye.

As the amount of lipase plays a role in the economical factor, the ratio of Novozym 435 to the starting material cytidine was evaluated. To investigate the enzyme effect, three different quantities were evaluated: 5.5, 12.7 and 22.5 wt% at a temperature of 95 °C (Figure 3). As expected, the conversion increases when using 12.7 wt% of lipase compared to 5.5 wt%. Using a higher concentration of 22.5 wt% decreases the yield due to enzyme aggregation formation [12]. The enzyme beads at the inside of these nuggets can, therefore, not react with the substrate due to the reduction in available active sites [37]. There is an increase in the yield until a reaction time of 30 min for every enzyme concentration. Therefore, an enzyme loading of 12.7 wt% was considered as optimum.

### 3.4. Effect of Reaction Temperature

**Figure 4:**
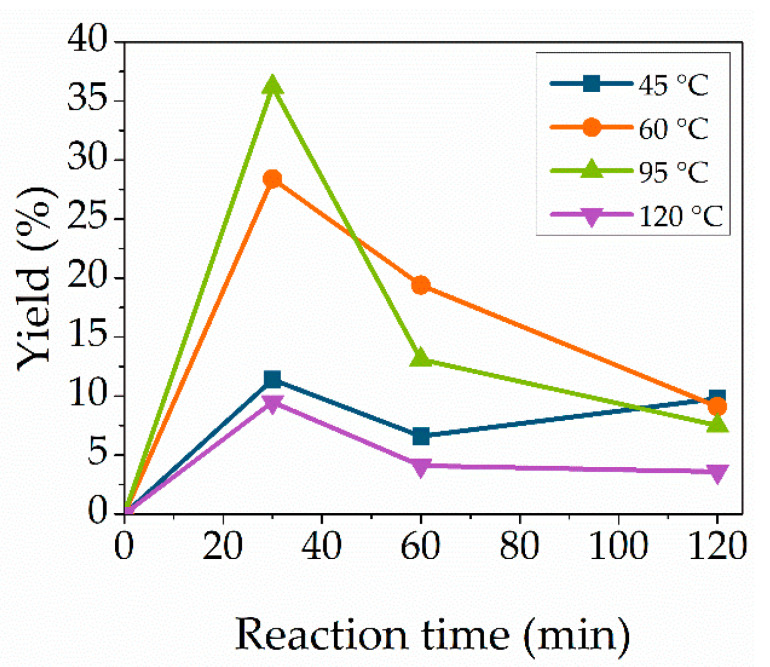
Effect of reaction temperature. Reaction conditions: cytidine (100 mg, 0.414 mmol), Novozym 435 (12.7 wt%), 1:35 molar ratio vinyl methacrylate at temperatures of 45 °C (blue), 60 °C (orange), 95 °C (green) or 120 °C (purple). Lines are included as a guide to the eye.

Temperature is one of the most crucial parameters in enzymatic catalyzed reactions. Reaction temperatures were varied between 45 °C and 120 °C to synthesize the cytidine-based monomer (Figure 4). High temperatures were chosen, as Novozym 435 is tolerant towards high temperature and is still active at temperatures higher than 90 °C [29]. The used substrate vinyl methacrylate exhibits a boiling temperature of 112 °C. Applying microwave irradiation enables us to heat chemicals above their usual boiling temperature due to increased pressure inside the reaction vessel and to the energy input [38]. The optimal temperature of Novozym is declared as between 40 °C and 65 °C. However, the yield increased with the rise in the temperature to a maximum yield of 36.2% at 95 °C, but lowered with a further rise to 120 °C. The color of the reaction mixture changed from clear to yellow at a temperature of 120 °C. The yield reduction at increased temperature is related to enzyme deactivation at high temperatures. The same trend was observed with lower enzyme and substrate concentrations. In addition, increasing the temperature leads to an increased reaction rate, resulting in a faster formation of by-products. The optimal reaction temperature with the highest conversion was at 95 °C.

### 3.5. Effect of Reaction Time

**Figure 5:**
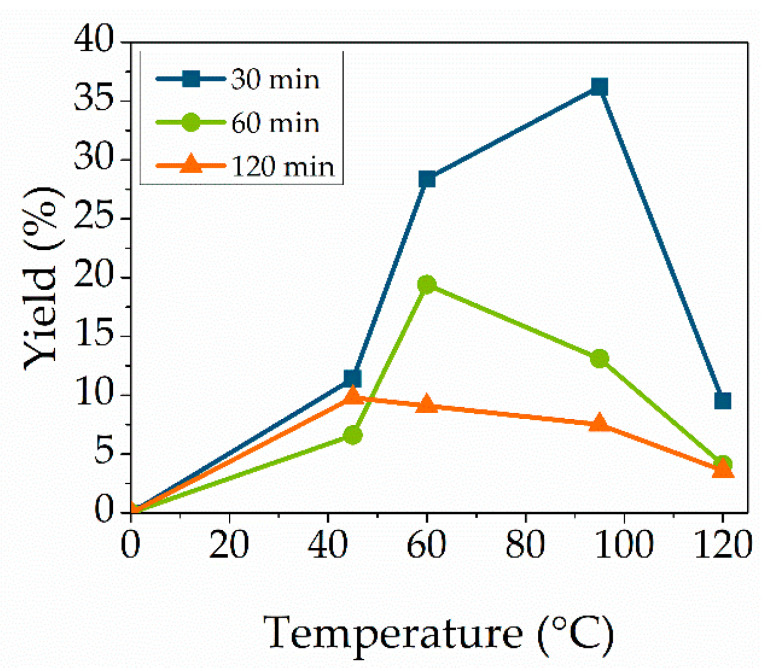
Effect of reaction time. Reaction conditions: cytidine (100 mg, 0.414 mmol), Novozym 435 (12.7 wt%), 1:35 molar ratio vinyl methacrylate at reaction times of 30 min (blue), 60 min (green) or 120 min (orange). Lines are included as a guide to the eye.

The effect of different reaction times was investigated. The reaction times were 10, 20, 30, 45, 60, 120 and 300 min (Figure 5). Reactions with reaction temperature of 45 °C, 60 °C and 95 °C all showed a yield maximum at 30 min. Shorter and longer reaction times resulted in lower yields. This observation might be explained by the equilibrium of this lipase-catalyzed transesterification as methacrylic acid could be detected in ^1^H NMR spectroscopy analysis. Longer reaction times lead to more undefined by-products according to TLC, HPLC and NMR spectroscopy analysis. Comparing these observations with previously described lipase-catalyzed (trans)esterification resulted in an opposite type of behavior [29,30,39]. While the yields in the literature reached a plateau at long reaction time, the yields decreased after a yield maximum. Initial attempts to explain this discrepancy were based on the formation of acetaldehyde, which results from tautomerization of the resulting vinyl alcohol when vinyl methacrylate is used as the substrate. In the presence of acetaldehyde, deactivation of the enzyme might occur [40]. Since, here, it is a closed reaction system, acetaldehyde cannot evaporate from the microwave reaction vessel, despite the low boiling temperature. To confirm this assumption, several experiments were repeated with a dissimilar substrate. Utilization of methyl methacrylate instead of vinyl methacrylate in the enzyme-catalyzed synthesis, likewise, displayed a decrease in yield with increased reaction times. Based on this result, the determining factor might be the combination of the starting material cytidine with Novozym 435 under microwave irradiation. In addition, cytidine bears a nucleophilic amino group, which can interfere with the reaction. Based on this result, the presence of acetaldehyde in previous experiments plays a more minor role than assumed. Rather, the discrepancy in the results compared with those in the literature is related to the combination of Novozym 435 with the starting material cytidine upon microwave irradiation. Thus, cytidine bears a nucleophilic amine group, which can interfere with the outcome of the reaction.

### 3.6. Effect of Molar Ratio

**Figure 6:**
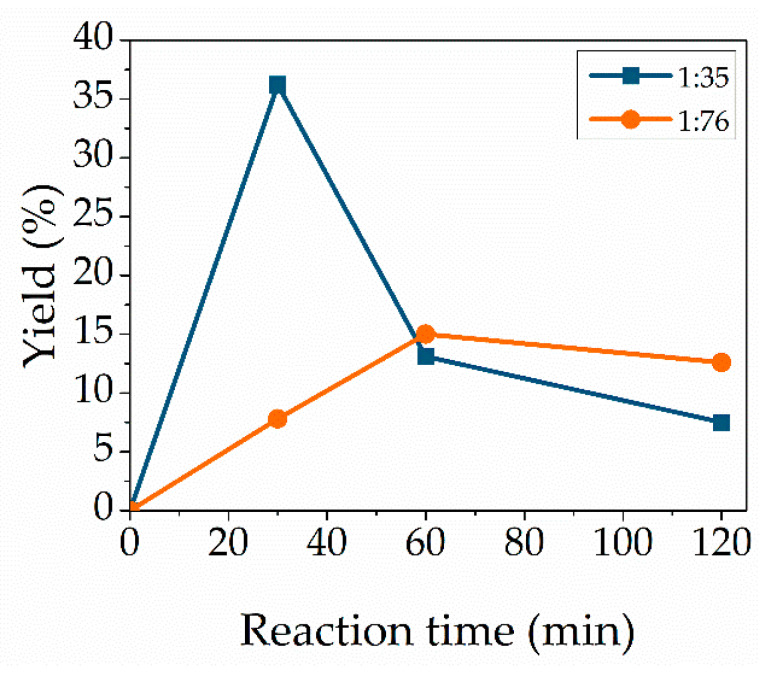
Effect of molar ratio. Reaction conditions: cytidine (100 mg, 0.414 mmol), Novozym 435 (12.7 wt%), 95 °C, vinyl methacrylate with a molar ratio of 1:35 (blue) or 1:76 (orange). Lines are included as a guide to the eye.

High molar ratios of the substrate were necessary as a certain volume is necessary for the microwave sensor and to prevent reverse reaction of the transesterification equilibrium. To evaluate the influence of the amount of the substrate vinyl methacrylate, the molar ratio to cytidine was changed from 1:35 to 1:76 (Figure 6). This obviously high excess of vinyl methacrylate showed first a yield decrease at a reaction time of 30 min compared to 1:35 but increases after a reaction time of 1 h and 2 h. After reaching the yield maximum, the yield decreases as well. This decrease in yield indicates enzyme inhibition when using high molar ratios of substrates. The active sites in the enzymes are inhibited due to the interaction between lipase and substrate, which leads to the formation of enzyme–substrate complexes [41]. Using a high excess of vinyl methacrylate can promote the formation of methacrylic acid, leading to a yield decrease.

### 3.7. Recyclability

The advantage of using immobilized enzymes for reactions is the reusability. Due to the strong support to the polymer, Novozym 435 can be easily filtrated from the reaction mixture. After washing and drying, the immobilized lipase is ready to be reprocessed. The reuse cycle is limited, as stirring and handling can cause physical damage. To verify the recyclability, the reaction with recycled Novozm 435 was compared with previously unused enzyme. Using the same reaction conditions of 95 °C and 2 h, the yield decreased from 7.5% to 1.7%. These results led to the statement that a reuse of Novozym 435 for the enzyme-catalyzed, microwave-assisted synthesis of 5′-*O*-methacryloylcytidine is possible; an optimized purification method is necessary.

## 4. Conclusions

The enzymatic microwave-assisted synthesis of 5′-*O*-methacryloylcytidine was optimized by using variable reaction conditions. Therefore, the impact of changing the enzyme and substrate concentration, reaction temperature and reaction time was observed. The optimum yield of 36% was obtained with a reaction temperature of 95 °C and time of 30 min, with a 12.7 wt% enzyme concentration and a 1:35 molar ratio for the substrate vinyl methacrylate. Vinyl methacrylate proved to be the preferable substrate compared to methyl methacrylate due to the poorer leaving group methanol. Higher enzyme concentration led to decreased yields, as the active sites are blocked due to aggregate formation. Increasing the reaction temperature and reaction time lowered the conversion due to enzyme deactivation and formation of undefined by-products. Using a higher molar ratio of 1:76 of vinyl methacrylate resulted in an inhibition of the active sites of the enzymes by forming strong enzyme–substrate complexes. With an optimized purification method, the used Novozym 435 can be reused and is, therefore, one step closer to green chemistry. 

## Data Availability

The main data presented in this study are available in the Supplementary Material.

## Supplementary Materials

The following supporting information can be downloaded at: https://www.mdpi.com/article/10.3390/molecules27134112/s1, Figure S1: ^1^H NMR of 5′-*O*-methacryloylcytidine; Figure S2: ^13^C NMR of 5′-*O*-methacryloylcytidine; Figure S3: 2D COSY NMR of 5′-*O*-methacryloylcytidine; Figure S4: 2D HSQC NMR of 5′-*O*-methacryloylcytidine; Table S1: Effect of the substrate choice. Reaction conditions: 12.7 wt% Novozym 435, 95 °C, 1:35 cytidine to substrate molar ratio; Table S2: Effect of enzyme concentration with 1:35 molar ratio vinyl methacrylate; Table S3: Effect of reaction temperature with a vinyl methacrylate molar ratio of 1:35; Table S4: Effect of reaction time with a 1:35 molar ratio of vinyl methacrylate; Table S5: Effect of molar ratio at a reaction temperature of 95 °C.

## Sample Availability

Samples of the compounds are available from the authors.
