# Supplementary material for: Microwave-Assisted Synthesis of 5′-O-methacryloylcytidine Using the Immobilized Lipase Novozym 435
Source: Molecules. 2022 Jun 26;27(13):4112. doi: 10.3390/molecules27134112 (PMC9268227; doi:10.3390/molecules27134112)
Supplement: Supplementary file 1 [file molecules-27-04112-s001.zip › molecules-1745867-supplementary.pdf]

Microwave-assisted synthesis of 5′-*O*-methacryloylcytidine using the immobilized lipase Novozym 435

Sany Chea [1,2], Khac Toan Nguyen [1,3] and Ruben R. Rosencrantz [1,*]

[1] Fraunhofer Institute of Applied Polymer Research, Biofunctionalized Materials and (Glyco)Biotechnology, Geiselbergstr. 69, 14476 Potsdam
[2] University of Potsdam, Institute of Chemistry, Chair of Polymer Materials and Polymer Technologies, Karl- Liebknecht- Str. 24-25, 14476 Potsdam
[3] Technical University of Berlin, Institute of Chemistry, Str. des 17. Juni 115, 10623 Berlin
* Correspondence: ruben.rosencrantz@iap.fraunhofer.de; Tel.: +49 331 568 3203

Figure S1. ^{1}H NMR of 5'-*O*-methacryloylcytidine. 1
Figure S2. ^{13}C NMR of 5'-*O*-methacryloylcytidine. 2
Figure S3. 2D COSY NMR of 5'-*O*-methacryloylcytidine. 2
Figure S4. 2D HSQC NMR of 5'-*O*-methacryloylcytidine. 3
Table S1. Effect of the substrate choice. Reaction conditions: 12.7 wt% Novozym 435, 95 °C, 1:35 cytidine to substrate molar ratio. 3
Table S2. Effect of enzyme concentration with 1:35 molar ratio vinyl methacrylate. 3
Table S3. Effect of reaction temperature with a vinyl methacrylate molar ratio of 1:35. 4
Table S4. Effect of reaction time with a 1:35 molar ratio of vinyl methacrylate. 5
Table S5. Effect of molar ratio at a reaction temperature of 95 °C. 5

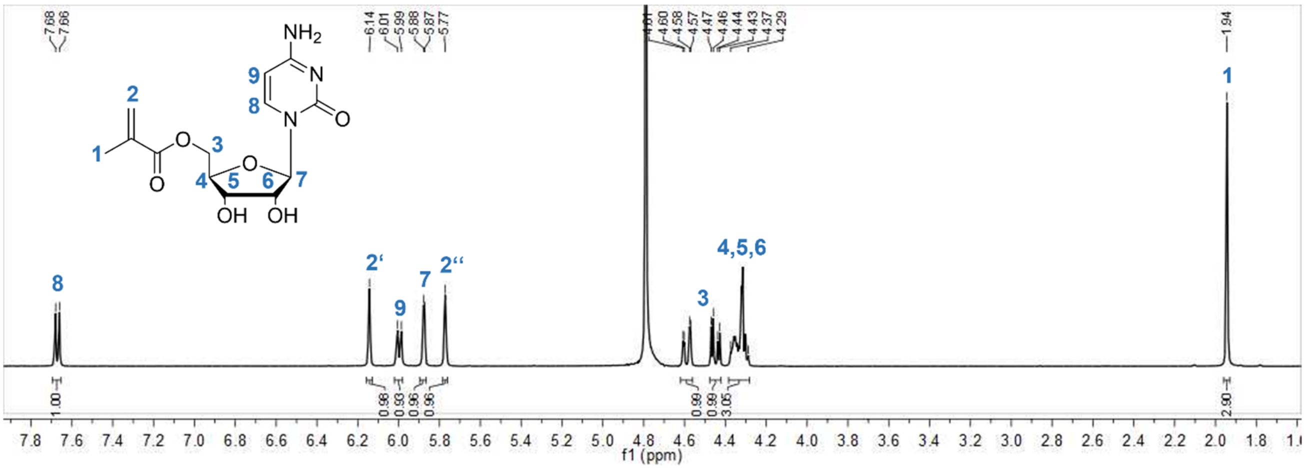

Figure S1. ^{1}H NMR of 5'-*O*-methacryloylcytidine.

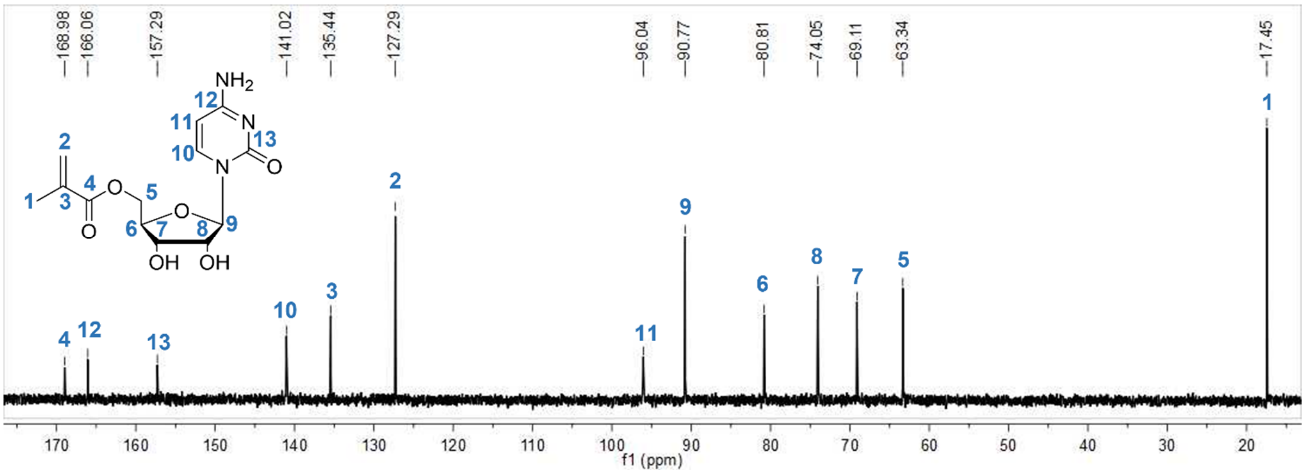

Figure S2. ^{13}C NMR of 5'-*O*-methacryloylcytidine.

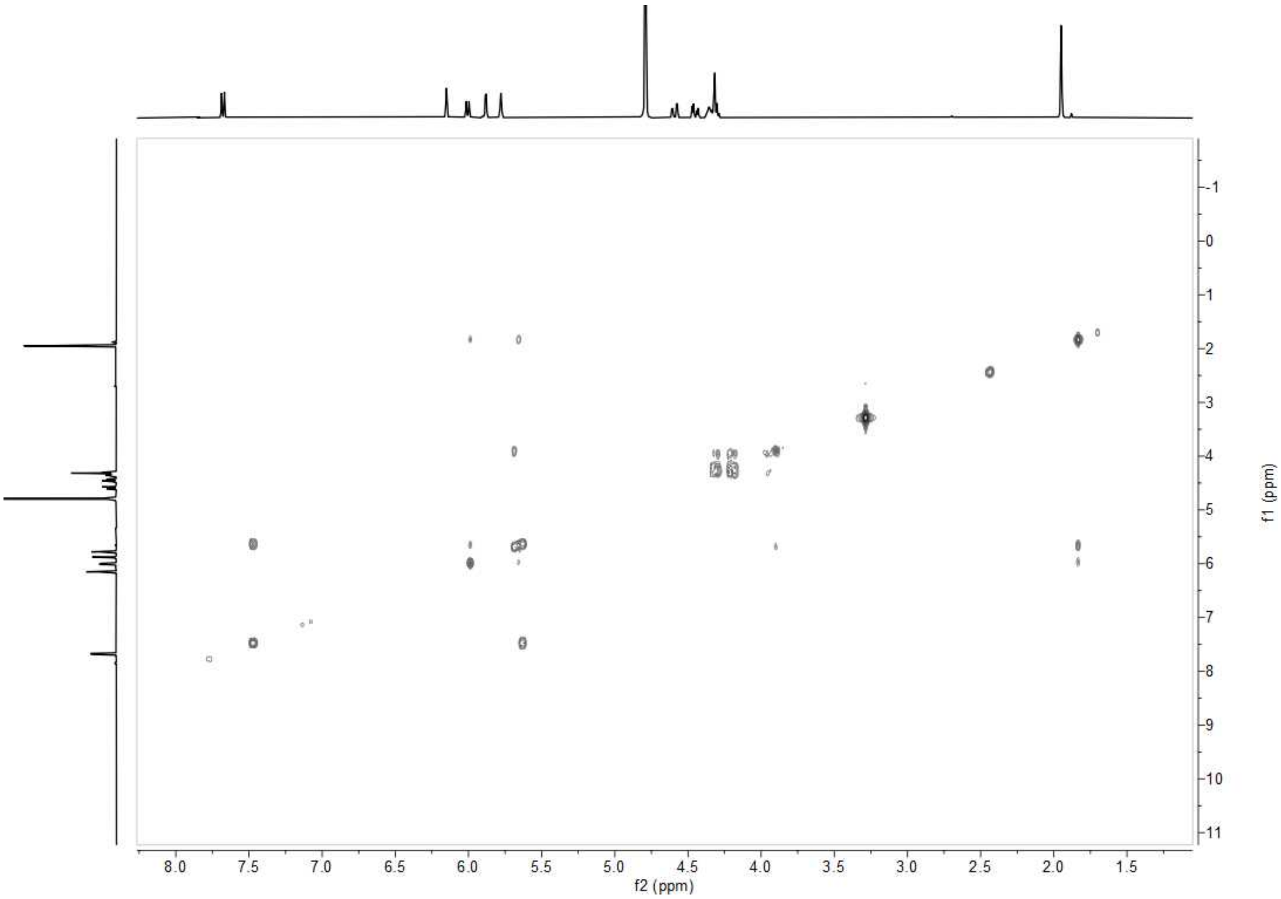

Figure S3. 2D COSY NMR of 5'-*O*-methacryloylcytidine.

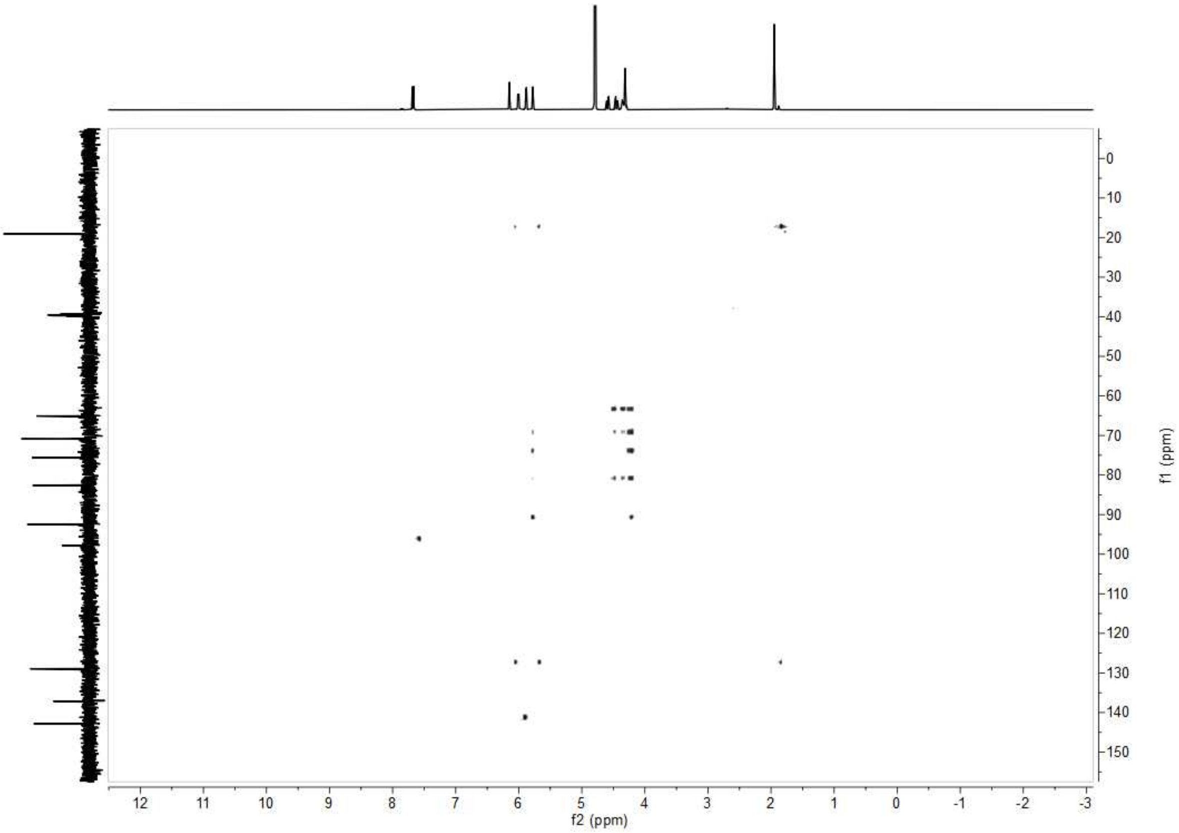

Figure S4. 2D HSQC NMR of 5'-*O*-methacryloylcytidine.

Table S1. Effect of the substrate choice. Reaction conditions: 12.7 wt% Novozym 435, 95 °C, 1:35 cytidine to substrate molar ratio.

Substrate	Time (min)	Yield (%)
Methyl methacrylate	30	9,4
	60	14,1
	120	7,2
Vinyl methacrylate	30	36,2
	60	13,1
	120	7,5

Table S2. Effect of enzyme concentration with 1:35 molar ratio vinyl methacrylate.

Concentration of lipase (wt%)	Time (min)	Temperature (°C)	Yield (%)
5.5	30	95	7.2
		60	2.8

		45	2.4
	60	95	7.8
	120	95	4.2
12.7	30	95	36.2
		60	28.4
		45	11.4
	60	95	13.1
	120	95	7.5
22.5	30	95	21.2
	60	95	11.0
	120	95	9.9

Table S3. Effect of reaction temperature with a vinyl methacrylate molar ratio of 1:35.

**Temperature (°C)**	**Concentration of lipase (wt%)**	**Time (min)**	**Yield (%)**
45	5.5	30	2.4
	12.7	30	11.4
		60	6.6
		120	9.8
60	5.5	30	2.8
	12.7	30	28.4
		60	19.4
		120	9.1
95	5.5	30	7.2
	12.7	30	36.2
		60	13.1
		120	7.5
120	12.7	30	9.5
		60	4.1
		120	3.6

Table S4. Effect of reaction time with a 1:35 molar ratio of vinyl methacrylate.

Time (min)	Concentration of lipase (wt%)	Reaction temperature (°C)	Yield (%)
10	12.7	95	11.6
20	12.7	95	19.9
30	5.5	95	7.2
	12.7	45	11.4
		60	28.4
		95	36.2
		120	9.5
	22.5	95	21.2
45	12.7	95	17.2
60	5.5	95	7.8
	12.7	45	6.6
		60	19.4
		95	13.1
		120	4.1
	22.5	95	9.9
120	5.5	95	4.2
	12.7	45	9.8
		60	9.1
		95	7.5
		120	3.6
	22.5	95	7.5
300	12.7	95	6.4

Table S5. Effect of molar ratio at a reaction temperature of 95 °C.

Substrate molar ratio	Enzyme concentration (wt%)	Time (min)	Yield (%)
1:35	12.7	30	36.2
		60	13.1
		120	7.5

		300	6.4
1:76	12.7	30	7.8
		60	15
		120	12.6
	22.5	120	17.3
		300	4.8
